# Recent Advances in Understanding the Role of Cartilage Lubrication in Osteoarthritis

**DOI:** 10.3390/molecules26206122

**Published:** 2021-10-11

**Authors:** Yumei Li, Zhongrun Yuan, Hui Yang, Haijian Zhong, Weijie Peng, Renjian Xie

**Affiliations:** 1 Key Laboratory of Prevention and Treatment of Cardiovascular and Cerebrovascular Diseases, Ministry of Education, Gannan Medical University, Ganzhou 341000, China; Yumei.Li@gmu.edu.cn (Y.L.); Yanghui_2512@gmu.edu.cn (H.Y.); hjzhong2007@gmu.edu.cn (H.Z.); 2 School of Basic Medicine, Gannan Medical University, Ganzhou 341000, China; 3 School of Materials Science and Engineering, South China University of Technology, Guangzhou 510641, China; zryuan1997@163.com; 4 National Engineering Research Center for Tissue Restoration and Reconstruction, South China University of Technology, Guangzhou 510006, China; 5 Jiangxi Province Key Laboratory of Biomaterials and Biofabrication for Tissue Engineering, Gannan Medical University, Ganzhou 341000, China; 6 School of Medical Information Engineering, Gannan Medical University, Ganzhou 341000, China

**Keywords:** articular cartilage, osteoarthritis, boundary lubrication, chondrocytes, shear stress

## Abstract

The remarkable lubrication properties of normal articular cartilage play an essential role in daily life, providing almost frictionless movements of joints. Alterations of cartilage surface or degradation of biomacromolecules within synovial fluid increase the wear and tear of the cartilage and hence determining the onset of the most common joint disease, osteoarthritis (OA). The irreversible and progressive degradation of articular cartilage is the hallmark of OA. Considering the absence of effective options to treat OA, the mechanosensitivity of chondrocytes has captured attention. As the only embedded cells in cartilage, the metabolism of chondrocytes is essential in maintaining homeostasis of cartilage, which triggers motivations to understand what is behind the low friction of cartilage and develop biolubrication-based strategies to postpone or even possibly heal OA. This review firstly focuses on the mechanism of cartilage lubrication, particularly on boundary lubrication. Then the mechanotransduction (especially shear stress) of chondrocytes is discussed. The following summarizes the recent development of cartilage-inspired biolubricants to highlight the correlation between cartilage lubrication and OA. One might expect that the restoration of cartilage lubrication at the early stage of OA could potentially promote the regeneration of cartilage and reverse its pathology to cure OA.

## 1. Introduction

Articular cartilage is an avascular, aneural and alymphatic connective tissue (which determines its very poor self-recovery ability) lining the bone ends of diarthrodial joints [1]. Combined with excellent load-bearing capacities, this cushion of articular cartilage, especially the outer surface of cartilage, provides extremely low friction with a friction coefficient as low as 10^−3^ under a wide range of physiological pressures (even up to 100 atm) to maintain daily movements during a person’s lifetime [2,3,4]. Recently, researchers noticed that the increase of cartilage friction plays a determining role in initiating the most common degenerative joint disability disease, that is osteoarthritis (OA), which is mainly characterized by the progressive but irreversible degradation of articular cartilage [5,6,7,8,9]. Briefly, aging-related changes or lesions usually lead to the compromisation of the outer surface of cartilage, which subsequently causes an increase of the friction coefficient. Chondrocytes, as the only cell type in cartilage, in return, up-regulate the secretion of the cartilage-degrading enzymes, such as matrix metalloproteinases (MMPs) and a disintegrin and metalloproteinase with thrombospondin motifs (ADAMTS) to degrade the type II collagen and aggrecan (the main components of cartilage matrix), respectively. Moreover, the cartilage degradation fragments are phagocytosed cells (such as macrophage and synovial fibroblasts) to inflame the synovium, promoting the production of MMPs and ADMATS to break down cartilage and deteriorate lubrication [10,11,12,13,14,15]. In this way, a positive feedback loop is formed due to the mutually reinforcing effect of increased friction and secretion of degradation enzymes, resulting in the progressive till total degradation of articular cartilage. Early, moderate, and late stages of OA can be classified mainly based on the degree of cartilage degradation. It is thought the breakdown of the type II collagen network initiates the point where the OA is considered irreversible [16,17,18]. Is it possible to modify and prevent the disease at its early stage especially considering the role of cartilage lubrication?

Among the population over 60 years old, 9.6% of men and 18.0% of women have OA symptoms, which made OA a serious global disease defined by the Osteoarthritis Research Society International in 2016 [19]. Cell-based therapies or cartilage tissue engineering for cartilage repair or regeneration have made significant advances recently [20,21], such as the adhesive peptide-based 3D scaffolds for cell culture [22], however, considerable efforts are still required for the tribological properties and durability of the neocartilage before clinical translation. Currently, the main nonsurgical options for OA treatment before the end of OA include using analgesics, anti-inflammatory drugs (such as acetaminophen), inhibitors (such as cyclooxygenase), or articular injection of hyaluronan and corticosteroids [23,24]. However, these nonsurgical options usually are highly controversial due to the nonuniversal effects when compared to that in state-of-the-art placebo-controlled [25,26]. Therefore, it is highly important to shed light on the remarkable lubrication of cartilage and the correlation between lubrication and cartilage regeneration, with the aim to improve the understanding of OA and encourage the development of approaches to alleviating and even treating it.

Intense research efforts have been made to deciphering the molecular mechanisms of cartilage lubrication since the 1930s [27]. A brief history of cartilage lubrication and the potential of injectable formulations to treat OA have been presented recently [18,28,29]. In this review, starting from the articular cartilage, we focus on discussing the boundary lubrication mechanism of cartilage, following by the shear-associated metabolism of chondrocytes, which could be fully taken advantage of to trigger cartilage regeneration, and finally, we review the recent biolubricants inspired by cartilage lubrication, a common theme of these inspired biolubricants is to resurface and restore the boundary lubrication of cartilage, and subsequently, alleviate the symptoms or even reverse the pathological progression of OA by promoting the regeneration of cartilage.

## 2. Articular Cartilage

A joint is a place where two or more bones meet, allowing the skeleton to move. Usually, joints differ in shape and structures according to the required movement and load, so we focus on discussing the diarthrosis knee joints. The knee joint consists of a joint capsule, ligaments, synovium, and the articular cartilage lining the ends of the opposing bones [1]. Synovial fluid within the capsule provides lubrication and nutrition, while the synovial membrane, a sac-like structure, surrounds the joint cavity and synovial fluid [30]. In daily life, it’s essential to maintain the normal structure of the synovial joint.

### 2.1. Structure and Components of Articular Cartilage

Articular cartilage (also referred to as hyaline cartilage) is a highly hydrated glassy connective tissue comprised of chondrocytes (the only cell type embedded within cartilage) and the extracellular matrix (ECM) which is secreted and maintained by chondrocytes [1,31]. The ECM is predominantly composed of type II collagen bundles, negatively charged proteoglycans, non-collagenous proteins, water, and ions (primarily Na^+^ and Cl^−^). 11 types of collagens could be found in articular cartilage. Among them, type II collagen, representing 90–95% of all collagens in ECM and accounting for 60% of the dry weight of articular cartilage, forms a crosslinked core network, to enable the cartilage tensile and shear strength [32,33,34].

The type II collagen fibril networks interweave with proteoglycans (such as aggrecans), the second most abundant macromolecules in articular cartilage, which contribute to the lubrication and load-bearing properties of cartilage due to their strong hydration [12]. Proteoglycans are proteins covalently attached to glycosaminoglycans (long repetitive dimers of hexosamine and uronic acid). The most prevalent and largest in size of proteoglycans in articular cartilage are aggrecans, representing a bottle-brush structure with a polypeptide as backbone and chondroitin sulfate and keratan sulfate as the side chains. Usually, there are over 100 chondroitin sulfate and 20–40 keratan sulfate chains in one aggrecan molecule [35,36]. Therefore, the aggrecans are highly sulfated and negatively charged conferred by the sulfate groups in their side chains. These negative charges attract large water molecules to further strengthen the cartilage matrix. Hyaluronic acid or hyaluronan (HA), the only non-sulfated glycosaminoglycan, is built by the repeated dimers of b-D-(1,4)-N-acetylglucosamine and b-D-(1–3) glucuronic acid with a molecular weight up to 6 MDa [37]. HA and aggrecan form an extensive aggregate, comprising of a central HA to which about 100 aggrecan molecules are non-covalently attached via the link protein, thereby stabilizing this aggregation [38]. These aggregates further bind to the type II collagen fibers and have been demonstrated to play a significant role in cartilage lubrication (will be discussed later). The other proteoglycans could be found in the backbone of articular cartilage including biglycan, decorin, fibromodulin, lumican and perlecan. Non-collagenous proteins mainly include fibronectin, cartilage oligomeric matrix protein (COMP), laminin, chondronectin et al. [39,40].

**Figure 1:**
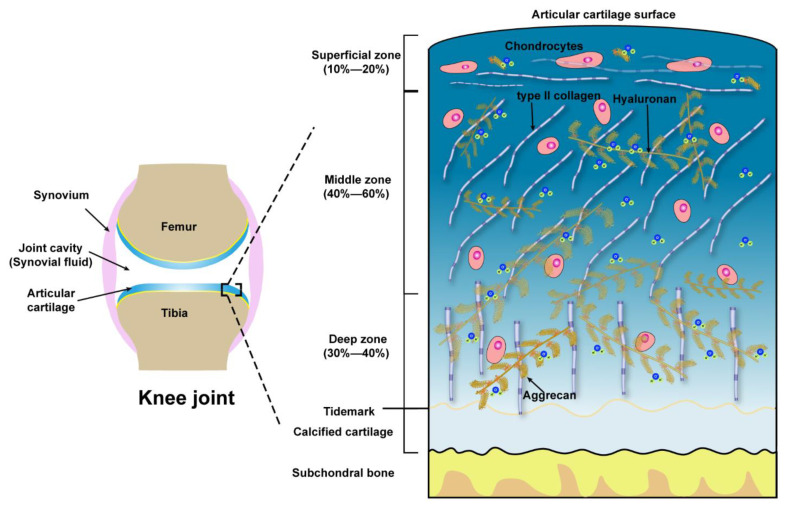
Schematic illustration of the knee joint and the structure, compositions of articular cartilage from the surface to subchondral bone.

Microscopically, three zones of the articular cartilage can be distinguished, that is the superficial zone (also referred to as the lamina splendens), the middle or transitional zone and the deep zone, as shown in Figure 1. These three zones of cartilage exhibit heterogeneity in the composition of ECM, which is reflected in the organization of collagen, size, phenotype, and metabolic activity of chondrocytes [41]. The superficial zone lies in the outer surface of articular cartilage, constitutes 10–20% of the full thickness of adult cartilage, characterized by two aspects, one is the type II collagen fibers, with a diameter of 30–35 nm, which are densely arranged and parallel to the articular surface, the other one is the long axis of flat and ellipsoidal chondrocytes parallel to the surface of the cartilage. Normally, the lubricating molecules are within the superficial zone. The middle zone is constituted of 40–60% of the thickness of total cartilage, the chondrocytes, exhibiting round or rectangular shape, are randomly distributed with their long axis perpendicular to the cartilage surface. The fibrils of type II collagen form an oblique transitional network and appear as arcades. The deep or radial zone constitutes the last 20–30% of the thickness of the cartilage. The shape of chondrocytes is round, the fibrils of type II collagen, with the largest diameter (40–80 nm), are perpendicular to the cartilage surface. The predominant biomechanical properties of the main three different zones of articular cartilage are summarized in Table 1.

### 2.2. Mechanotransduction of Chondrocytes

Mechanotransduction refers to the process of sensing and converting mechanical signals into biochemical signals to regulate cellular activities [52]. Located on the joint surfaces, a range of static and dynamic stresses (standing, walking, and jogging) are applied on articular cartilage. It is well documented the metabolism of chondrocyte is strongly regulated by the normal stress (compression), static loads were shown to be detrimental to the anabolic processes (biosynthesis of type II collagen and proteoglycans) while oscillatory loads with moderate frequencies and amplitudes (compression strain under 20%) have been shown to effectively promote the matrix accumulation and decrease the secretion of TNF- a and IL-6, which contribute to the degradation of matrix [53,54,55,56].

**Figure 2:**
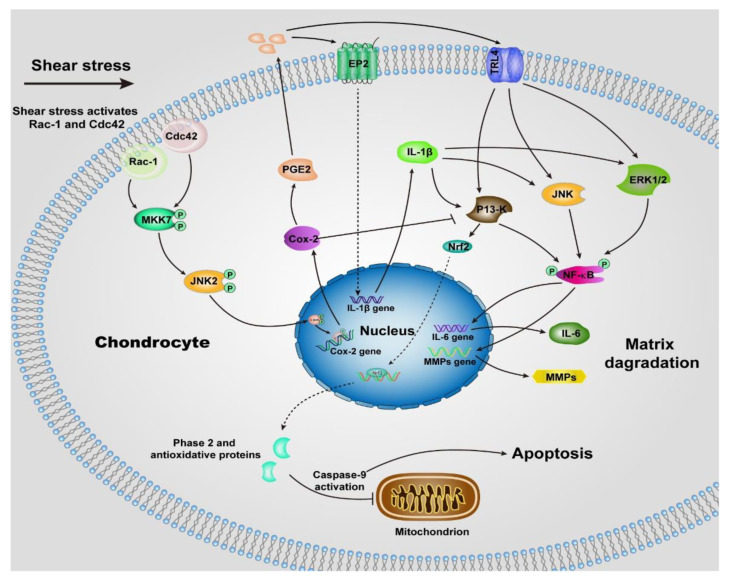
Schematic of shear-induced cartilage matrix degradation and apoptosis of chondrocytes. High shear stress activates the Rac-1/Cdc42, which then transactivates MKK7 to regulate JNK2 activation, and this, in turn, triggers c-Jun phosphorylation which induces the overexpression of Cox-2. Cox-2 suppresses the activity of P13-K, which represses Nrf-2 to decrease the antioxidant capacity to permit disruption of the integrity of mitochondrial, activation of caspase-9, and the apoptosis of chondrocytes. The expression of Cox-2 also triggers the expression of PGE2, as well as the concomitant downstream expression of receptor EP2, as a result, IL-1b is rapidly and sustainably synthesized. Moreover, up-regulation of TLR4 due to high shear stress activates ERK1/2, P13-K and JNK pathways, which is also activated by IL-1b, then regulates NF-kB-dependent IL-6 and MMP synthesis. Abbreviations: Cyclooxygenase-2 (Cox-2), mitogen-activated protein kinase 7 (MKK7), nuclear factor-kB (NF-kB), prostaglandin E2 (PGE2), Interleukin-1b (IL-1b), Interleukin-6 (IL-6), phosphatidylinositol 3-kinase (PI3-K), c-Jun N-terminal kinase 2 (JNK2), NF-E2 related factor 2 (Nrf2), matrix metalloproteinases (MMPs), toll-like receptor 4 (TLR4)**,** extracellular signal-regulated kinase (ERK1/2).

Unlike normal stress, shear stress gives rise to the shear strain of the cartilage, and then be transmitted to the chondrocytes, especially those within the superficial zone. Many previous studies suggested the shear stress activates chondrocytes and up-regulates the proinflammatory cytokines (TNF-a, and the family of interleukins) and MMPs [55,57], which elicit the degradation of cartilage, most of the underlying signaling pathways remain unclear, but we can summarize some of them in Figure 2 according to the previous reports [57,58,59,60,61,62,63]. The chondrocytes undergo a phenotypic switch to aberrantly express catabolic enzymes when the shear strain exceeds ≈1% estimated by Klein and Lin very recently under relative ideal circumstances considering the complexity of cartilage [64]. Moreover, the increased shear strain, or the shear stress or friction, induces chondrocyte apoptosis, which has been demonstrated by previous studies [65,66]. The mechanism regulating shear-mediated chondrocytes expression of IL-6 and MMPs and apoptosis is shown in Figure 2. The stimuli of high shear stress induce chondrocytes to express cyclooxygenase (Cox 2), which inhibits the activity of phosphatidylinositol 3-kinase (PI3-K), following decreases in antioxidant capacity to lead to chondrocyte apoptosis.

Collectively, favoring cartilage longevity requires dynamic normal stress combined with quite low or even zero shear stress. To maximize the regeneration of cartilage, the main way is to decrease the shear strain arising from the shear stress by reducing the friction coefficient of cartilage. Therefore, a scenario in which treating or healing OA at its early stage by restoring the lubrication of OA-damaged cartilage can be imagined.

### 2.3. Lubrication Mechanism of Articular Cartilage

The friction coefficients of normal articular cartilage can be as low as ~0.002–0.02 [64]. The lubrication properties of articular cartilage have drawn attention since the 1930s and many theories have been proposed to claim the mechanisms behind the ultra-low friction of cartilage. Over the development of these decades, one could simplify these theories into fluid film lubrication and boundary lubrication [8].

In the context of fluid film lubrication, an early and classical lubrication mechanism purported that a fluid film that completely separates the opposing surfaces to prevent direct contact between surfaces is formed during articulation, and this fluid film enables the low friction coefficient [67,68,69]. Considering the “biphasic” structure (solid phase of the matrix and the liquid phase of the water within the cartilage matrix), the models to elucidate the fluid film lubrication mechanism were also further developed. When cartilage surfaces slide past each other under compression, the interstitial fluid within the deformed cartilages, on one hand, is extruded out to form a film between the cartilage surfaces, on the other hand, the interstitial fluid is pressurized (due to the nature of incompressible fluid) to support the major normal load, and consequently reduces the proportion of the normal load that supported by the cartilage matrix [70,71]. Thus, the cartilage exhibits low friction. One question that should be noticed here is the pressure used in these related experiments was not very high (about 0.2 MPa, especially compared with that exerted on the cartilage in vivo), so the formed fluid film might be squeezed out in the case of high pressure. In this situation, boundary lubrication is proposed.

In the context of boundary lubrication, friction arises from the partial contact between the opposing cartilage surface asperities. The friction coefficient is relatively independent of the sliding speed and normal load and is dominated by the outer surface of the articular cartilage and the synovial fluid, especially the molecules within the synovial fluid [72,73]. The main components in the synovial fluid from the human joint include hyaluronic acid, albumin, globulin, lubricin, phospholipids, and glycosaminoglycans [74]. Among these components, lubricin and phospholipids are commonly found at the surface of cartilage at high concentrations.

Typically, three main different research approaches were shown to explore which component in synovial fluid is responsible for the ultra-low friction of articular cartilage: selectively enzyme-degraded specific ones or more components in synovial fluid or cartilage [75], or selectively added ones or more purified (or artificially synthesized) components to the lubricating bath [72] or direct measurement of the lubrication abilities of one or more components at the molecular level [76,77,78]. Radin et al. found no difference in the friction coefficient when HA was removed from the synovial fluid using hyaluronidase [79]. It was also found that the lubrication ability of HA was quite poor when confined to HA molecules under limited space [80]. Collectively, HA, on its own, cannot explain the remarkable lubrication of articular cartilage. Lubricin, also known as proteoglycan 4, has been reported to significantly reduce the friction coefficient of cartilage in vitro, and its deficiency induces chondrocyte (especially those embedded within the superficial zone) apoptosis in vivo by knocking out the PRG 4 gene [65]. Therefore, it is thought lubricin is a key lubricant for the boundary lubrication of articular cartilage. Molecularly, Zappone et al. found the friction coefficient of surfaces bearing lubricin was relatively low (μ = 0.02–0.04) at the pressure of about 6 atm, while increased to ≈0.2 under higher pressure [81]. Additionally, when constructing the surfaces bearing lubricin and HA layer by layer, the friction coefficients are still quite high compared with those found in articular cartilage, especially under the pressure at the level of a few atmospheres (atm.) [80]. Phospholipids, such as dipalmitoyl-phosphatidylcholine, phosphatidylethanolamine and sphingomyelin, become active upon binding with the calcium ions [82], have also been reported to bind to the cartilage surface and act as the boundary biolubricants. Many studies have shown a strongly absorbed active phospholipid layer that can exhibit extremely low friction coefficients under pressures up to hundreds of atm., which is near to the level of pressure the articular cartilage is exposed to in vivo [83,84]. However, when treated with phospholipase or added phospholipids (containing HA and lubricin), there was little change in the friction coefficients of the synovial fluid [72,85]. Overall, how the components in the synovial fluid enable the extremely low friction coefficient, especially under high pressure, has not yet been identified.

**Figure 3:**
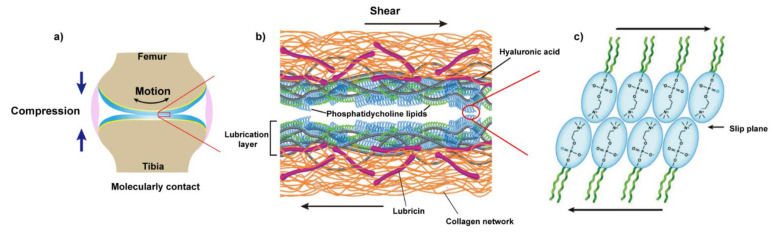
The schematic of boundary lubrication of articular cartilage. (**a**) Under high pressure, the outer surfaces of opposing articular cartilage were molecularly contacted. (**b**) The proposed structure of the lubrication layer on the outer surface of cartilage, exposing the phosphocholine groups of lipids at the interface to reduce friction via the hydration mechanism (Reprinted with permission from Ref. [86]. Copyright 2016 Annual Reviews INC); (**c**) Representation of the phospholipids at the interface of opposing cartilage. Plane (**c**), Reprinted with permission from Ref. [89]. Copyright 2015 American Chemical Society.

Recently, as shown in Figure 3, a new picture emerged where it is the synergy between the molecules in the synovial fluid, especially those mentioned above, that determines the lubrication of articular cartilage under severe joint loading [86,87]. Specifically, HA associates with aggrecan via the link protein to form a bottle-brush structure where HA serves as the backbone and aggrecan as the side chain. HA and lubricin also form a complex, which is physically trapped on the surface and contributes to effectively eliminate the wear damage of the cartilage. HA also shows a high affinity with phospholipids according to the previous report [88]. Thus, Klein et al. pointed to a scenario in which HA (alone cannot bind to cartilage surface) anchors at the outer surface of cartilage with the assistance of lubricin, then further complexes with the phospholipids to act as an effective boundary lubricant to enable the remarkable lubrication of articular cartilage at high pressure, via the hydration mechanism [89,90,91]. In this way, lubricin serves as a “carrier” between the HA, phospholipids, and the outer surface of the articular cartilage.

## 3. Biolubricants Inspired by the Cartilage Lubrication with the Aim to Treat OA

As mentioned above, a layer of complex formed by the synergy of synovial fluid components acts as boundary lubricants to reduce the friction between opposing articular cartilage surfaces, especially at high pressure, and effectively ensure the nearly frictionless motion of knee joints. However, disruption of the integrity of cartilage surface and alteration in the composition of synovial fluid compromise this boundary lubricant layer, initiates a cascade of biological and biomechanical events lead to dysfunction of boundary lubrication and then triggers the development of OA [92,93]. This association between the loss of lubrication layer at the outer surface of the articular cartilage surface and OA pathogenesis has been demonstrated in OA animal models [94,95]. In the context of the correlation between dysfunction of cartilage lubrication and the stimuli-response of chondrocytes, especially the increased secretion of catabolic matrix degraded enzymes and the decreased secretion of cartilage matrix, researchers have focused on alleviating and treating OA by restoring the lubrication properties of damaged or OA affected articular cartilage. Hydration lubrication is thought to be the dominant lubrication mechanism in biological systems, so highly hydrophilic monomers are usually the prior choice used to design biolubricants [90] so that a robust hydration layer to reduce the friction could be formed once the designed biolubricants resurface the cartilage. The typical biolubricants inspired by cartilage lubrication with the aim to treat OA developed recently are summarized in Table 2, most of them are described in detail in the following sections.

### 3.1. Natural Lubricants and Their Derivatives

**Figure 4:**
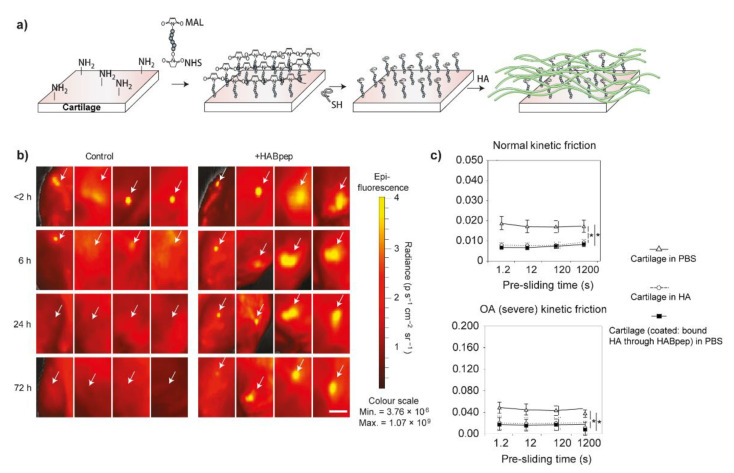
(**a**) Schematic process of cartilage-surface modification of peptide-polymer HABpep to arrest HA in the surrounding synovial fluid. MAL-PEG-NHS, as a crosslinker, was firstly reacted with the exposed primary amines in cartilage surface and then subsequently reacted with the thiolated HABpep by click reaction to prepare a coating that can recruit HA to the surface of the cartilage. (**b**) After modified the cartilage surface with HABpep, the HA molecules tagged with rhodamine (White arrows) were retained within the joint for 72 h post articular injection, whereas, the retention time of HA was only 6 h without this coating. Scale bar, 1 cm. (**c**) The kinetic friction coefficients of normal cartilage samples (upper) and severe OA cartilage samples (lower) with or without modification. Reprinted with permission from Ref. [96]. Copyright 2014 Springer Nature.

Hyaluronic acid, gives the synovial fluid high viscoelastic properties, was thought to ensure the lubrication of cartilage in the 1950s, especially when associated with significantly decreased viscoelasticity of synovial fluid from OA patients [111,112]. Therefore, articular injection of high molecular weight HA (usually above 1 MDa) has been used to alleviate the symptoms of OA clinically as viscosupplementation. However, the results are controversial due to the universal individual differences. The main reasons may be due to the short retention time in the joint cavity and the enzymatic degradation [96]. In this case, chemical modification and cross-linking are the main two options to try to bypass these two shortcomings. Elisseeff et al. developed a strategy to concentrate the HA to re-construct the boundary lubrication of OA-damaged cartilage by creating a HA-recruiting coating (called HABpep) on the outer surface of cartilage (Figure 4a) [96]. This method significantly increased the retention of HA in the joint and even improved the lubrication of OA-damaged cartilage to the level of healthy cartilage (Figure 4b,c). Based on these results, similarly, they further employed HA binding peptide-polymer systems (HABP2-8-arm PEG-COLBP) in vivo with the aim to reduce OA progression and treat OA. After localizing to the cartilage defects, these systems showed positive therapeutic results by decreasing the level of inflammatory factors and increasing the expression of aggrecan, and thus, slow down the cartilage degradation [97]. Recently, chondroitin sulfate was also used to be modified by HA binding peptide (used to recruit HA in synovial fluid) and type II collagen peptide (used to bind to the surface of cartilage) simultaneously, this library of peptide-modified chondroitin sulfate also resurfaced and then decreased the friction coefficients of articular cartilage without modification of the cartilage [98]. In addition to peptide modification, Zhang et al. grafted 2-methacryloyloxyethyl phosphorylcholine (MPC), a zwitterionic monomer with robust lubrication performance [113], to HA with low and high molecular weights (lHAMPC and hHAMPC) in order to enhance HA lubrication ability [99]. The friction coefficients of polystyrene microspheres versus silicon wafer lubricated by lHAMPC and hHAMPC aqueous solution were significantly decreased when compared with the control groups (water and HA solution). Additionally, HAMPC could upregulate the chondrocytes anabolic genes and downregulate the chondrocytes catabolic genes, especially the hHAMPC. However, the lubricating properties of these two polymers were not demonstrated by cartilage.

**Figure 5:**
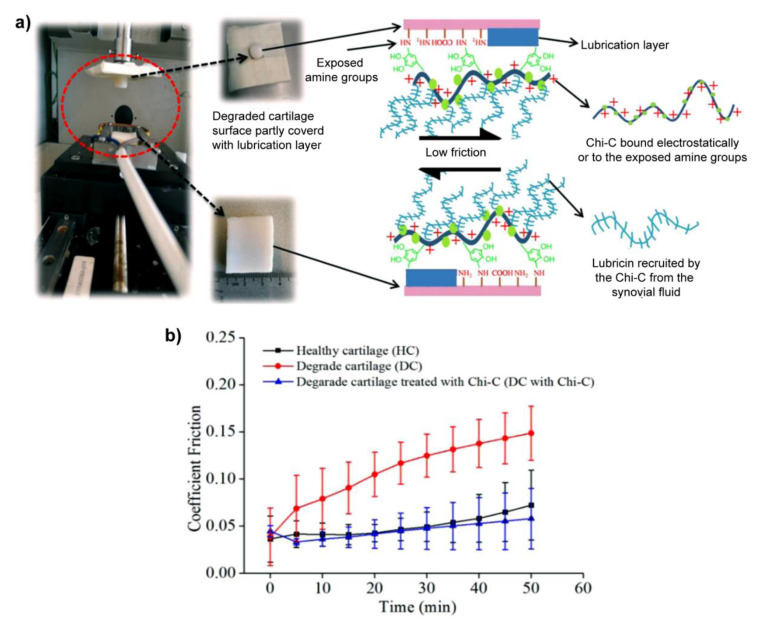
(**a**) Schematic of mucoadhesive biopolymer Chi-C bound to the surface of degraded cartilage and recruited lubricin to restore the lubrication layer to reduce the friction coefficients. The friction coefficients were measured by Universal Mechanic Tester 3 under the back-and-forth mode. The size of the bottom cartilage sample (extracted from the femur of a bovine knee joint) is 40 × 25 mm^2^ with a thickness of 5 mm, and the diameter of the top cylinder is 9 mm (extracted from the tibia of a bovine knee joint). (**b**) Friction coefficients of healthy, degraded, and Chi-C treated cartilage lubricated by bovine synovial fluid. Reprinted with permission from Ref. [100]. Copyright 2020 Elsevier B.V.

Very recently, recruiting the lubricin, a natural biolubricant, from the surrounding milieu emerged as another strategy to restore the lubrication of cartilage, as shown in Figure 5, Sharma et al. modified the chitosan with catechol (Chi-C) to prepare a mucoadhesive biopolymer, which can bind to the cartilage surface by electrostatic interaction and/or the chemical reaction between the oxidized derivative of catechol and amines exposed in degraded cartilage so that the sessile Chi-C recruited the lubricin (the oxidized catechol reacted with amines in lubricin) to restore the lubrication layer of damaged cartilage. Interestingly, this method reduced the friction coefficients of degraded cartilage to the level of healthy cartilage [100]. Additionally, they extended this thought to polycarbonate urethane, a biomaterial used for meniscus replacements, to reduce the friction coefficients when sliding against cartilage [114].

### 3.2. Fully-Synthetic Polymer Lubricants

Many fully synthetic biolubricants were designed and synthesized and exhibited remarkable lubrication at the molecular level measured by a very typical and sensitive technique, surface force balance, or surface force apparatus [115,116,117]. In this section, we only focus on discussing the fully synthetic biolubricants developed recently, which clearly claimed to restore the lubrication of cartilage.

**Figure 6:**
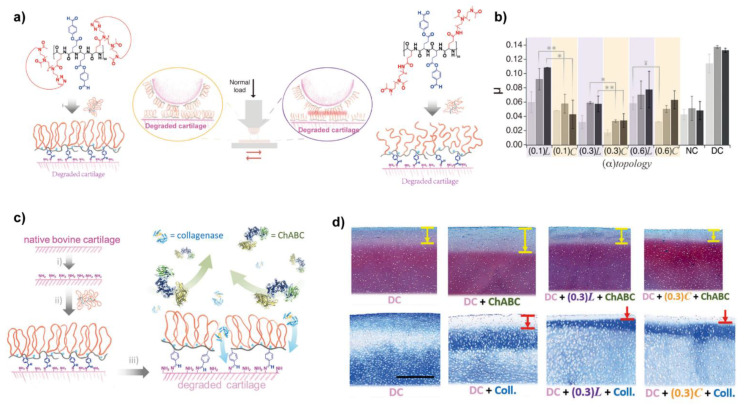
(**a**) Comparison of the chemical structure of the PGA-LPMOXA-HBA and PGA-CPMOXA-HBA and their schematic adsorption on the degraded cartilage; (**b**) The lubricating abilities of PGA-LPMOXA-HBA and PGA-CPMOXA-HBA with series of partial of PMOXA using degraded cartilage (DC) and normal cartilage (NC) under different pressures (light gray, 0.5 MPa; gray, 0.7 MPa; dark gray, 0.9 MPa). The friction coefficients were significantly reduced when replaced the LPMOXA by CPMOXA. (**) *p* < 0.05; (*) *p* < 0.01; (^¥^) *p* = 0.095; (**c**) Schematics of the derived lubrication film formed by PGA-CPMOXA-HBA protected the cartilage from enzymatic degradation. (i) ChABC (1 U/mL, 0.01% BSA in PBS), 1 hour, 37 °C; (ii) 0.1 mg/mL PGA-(α) CPMOXA-HBA in HEPES II, overnight; (iii) ChABC (1 U/mL, 0.01% BSA in PBS), 1 hour, 37 °C or collagenase (1.2 mg/mL in PBS), half an hour, 37 °C; (**d**) Safranin-O staining treated by PGA-LPMOXA-HBA (0.3 L) and PGA-CPMOXA-HBA (0.3 C). The arrows indicated the enzyme-penetrated depth. Scale bar, 500 μm. Reprinted with permission from Ref. [102]. Copyright 2018 Wiley-VCH.

Benetti et al. designed and synthesized a series of bottle-brush graft-copolymers where linear poly-2-methyl-2-oxazoline (LPMOXA) was alternatively tethered together with aldehyde-bearing hydroxybenzaldehyde (HBA) to the polyglutamic acid (PGA) (PGA-LPMOXA-HBA), as shown in Figure 6, these copolymers anchored on the surface of degraded cartilage via Schiff base between aldehyde and amino groups (presented in collagen), thus a boundary lubrication layer was formed and the part of PMOXA provided lubricity, the lubrication test showed the friction coefficients of cartilage resurfaced by PGA-LPMOXA-HBA were significantly reduced [101]. Additionally, due to the biopassivity provided by PMOXA, protein adsorption was prevented by the films of PGA-LPMOXA-HBA. Followingly, they replaced the LPMOXA with cyclic poly(2-methyl-2-oxazoline) (CPMOXA) to synthesis another series of biolubricants with different densities of side chains, called PGA-CPMOXA-HBA [102]. After chemosorbing on the surface of cartilage via Schiff bases, PGA-CPMOXA-HBA generated denser lubricious films due to the absence of chain ends of the exposed CPMOXA when compared with PGA-LPMOXA-HBA, so PGA-CPMOXA-HBA reduced the friction coefficients of treated cartilage even under the higher load (Figure 6b). Noteworthy, the films of PGA-CPMOXA-HBA effectively prevent the penetration of catabolic enzymes (MMPs and ChABC) and thus, reduced cartilage degradation up to 50% (Figure 6c,d).

Additionally, Lawrence et al. designed a library of bottle-brush copolymers with different size of backbone and side chains, and density of side chains to mimic lubricin. Highly hydrophilic polyethylene glycol (PEG) was tethered to polyacrylic acid (PAA) to build the biolubricant pAA-g-PEG, and a thiol terminus was anchored so that pAA-g-PEG could bind to the cartilage surface. These copolymers could resurface the cartilage within 39 min and then effectively lubricated the cartilage [103,105]. Recently, they further evaluated the lubrication of these copolymers using the cartilage harvested from surgery-induced rats 8 weeks after articular injection. There were no differences in the changes of friction coefficients in all treated groups, which may be due to the influence caused by the limited size of rat cartilage. However, the other histological results may demonstrate the correlation between restoring lubrication and attenuating the progression of OA [104].

### 3.3. Nanospheres or Nanoparticles and Nano (Micro) Gels

Nanospheres or nanoparticles and microgels modified with highly hydrated polymers were also developed as biomimetic lubricants recently. The major advantage of these biomimetic lubricants is to combine lubrication with drug-releasing so that they present dual functions. In recent two years, many such biomimetic lubricants emerged. Zhou et al. modified the polystyrene (PS) nanospheres with poly (3-sulfopropyl methacrylate potassium salt) (PSPMA) via atom transfer radical polymerization [106]. Another charged chitosan nanoparticles modified with hydrophilic sulfate groups via ring-open reaction and then loaded aspirin (an anti-inflammatory drug) were prepared as dual-functional lubricants to treat OA [107]. These series of biolubricants, combined with the previous analogous work [118,119,120], greatly decreased the friction coefficient due to the formed thick hydration layer around the nanospheres or nanoparticles. However, the potential of such biolubricants being used to treat OA requires further studies since they mainly focus on the lubrication properties.

**Figure 7:**
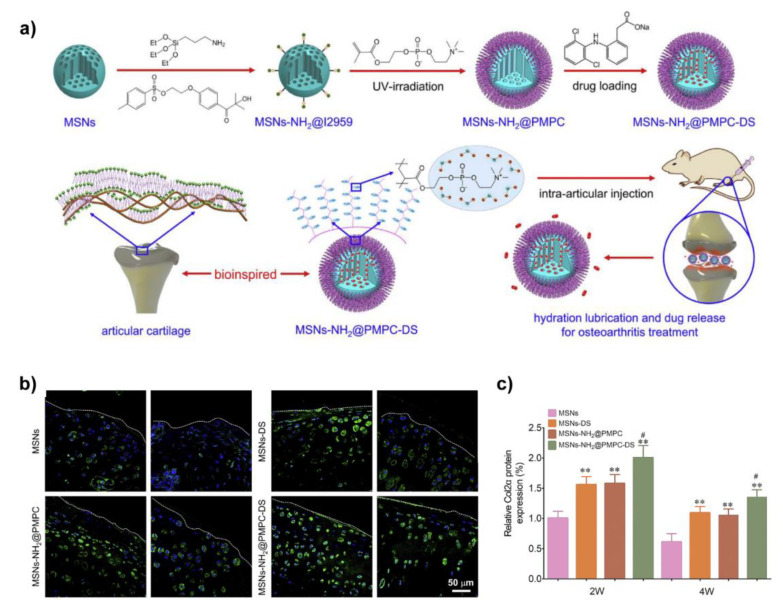
(**a**) Schematic of the preparation of MSNs-NH_2_@PMPC-DS with the aim to lubricate and treat OA; (**b**) Representative images of immunohistochemistry staining of the expression level of Col2α estimated by the surgery-induced OA rats; (**c**) The quantitative level of Col2α was obtained based on fluorescence intensity in Figure 7b, *n* = 5 and the data were presented as mean ± SD, ** *p* < 0.01, MSNs as the control group, ^#^ *p* < 0.05, MSNs-DS as the control group. Reprinted with permission from Ref. [108]. Copyright 2020 Elsevier.

From this point of view, as shown in Figure 7, Zhang et al. grafted PMPC onto the surface of mesoporous silica nanospheres (MSN) via photopolymerization to prepare biomimetic lubricated nanospheres (MSNs-NH_2_@PMPC), meanwhile, diclofenac sodium (DS), an anti-inflammatory drug, was encapsulated in MSNs-NH_2_@PMPC to prepare dual-functional (lubrication and drug delivery) biolubricants MSNs-NH_2_@PMPC-DS. They found MSNs-NH_2_@PMPC (10 mg/mL in aqueous) were adsorbed and formed a thin film on the surface of Ti6Al4V, and hence acted as a boundary lubrication layer to significantly reduce the friction coefficient when PE sphere pin sliding against Ti6Al4V lubricated by MSNs-NH_2_@PMPC solution. Further in vivo treatment of OA was evaluated after confirming the biocompatibilities of MSNs-NH_2_@PMPC and MSNs-NH_2_@PMPC-DS. The results indicated that the MSNs-NH_2_@PMPC-DS best inhibited the degradation of cartilage, and promoted the expression of type II collagen, which was attributed to the synergy of lubrication and drug delivery [108]. By replacing the PMPC with 3-dimethyl-[2-(2-methylprop-2-enoyloxy)ethyl]azaniumyl]propane-1-sulfonate polymer (pSBMA), the same dual-functional biomimetic lubricants were also prepared [109].

**Figure 8:**
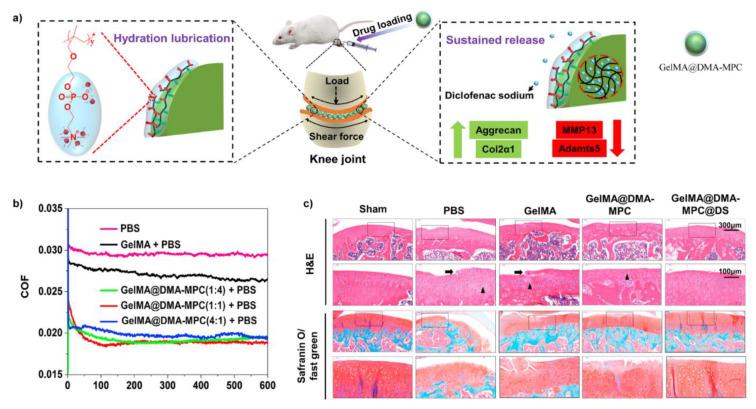
(**a**) Schematic of OA treatment by articular-injecting of dual-functional GelMA@DMA-MPC@DS with enhanced lubrication and sustained release of the drug; (**b**) The plots of friction coefficients versus time using PTFE pin and a silicon wafer as friction pairs; (**c**) Representative images of histological staining assay showed the disruption of cartilage with different treatments. The black arrow shows the fissures within cartilage, the black triangle indicates tissue cellularity with cloning. Reprinted with permission from Ref. [110]. Copyright 2021 KeAi publishing.

Very recently, considering such biolubricants were articularly injected into the joint of OA model rats, a new constructing method was reported in order to enhance the injectability [110]. As shown in Figure 8, Methacrylate gelatin (GelMA) hydrogel microspheres were firstly prepared by microfluidic fabrication, then copolymer polymerized by the monomer methacrylate dopamine (DMA) and MPC was absorbed onto the surface of GelMA to generate biolubricants GelMA@DMA-MPC via the robust adhesiveness of dopamine. Subsequently, DS was encapsulated in GelMA@DMA-MPC (GelMA@DMA-MPC@DS) to enable lubrication and drug-delivery simultaneously. As the core of GelMA@DMA-MPC was a microgel, the injectability was thought to be enhanced. The friction coefficients were most decreased by ~29.6% when PTFE sliding against silicon wafer. Additionally, when co-cultured with chondrocytes, it was found GelMA@DMA-MPC@DS contributed to protecting the chondrocytes from degeneration associated with inflammation by increasing the expression of type II collagen and down-regulating the level of MMP13 and ADAMTS5. Relatively excellent lubrication ability and biocompatibility permitted GelMA@DMA-MPC to be further investigated to examine the in vivo therapeutic effects in OA model rats. The in vivo results revealed GelMA@DMA-MPC@DS delayed the degradation of cartilage and showed the best therapeutic effects.

One may notice that the lubrication performance of this series of biomimetic lubricants was evaluated by some specific polymers (PDMS, PTFE, PE) or alloys (Ti6Al4V) rather than cartilage though it was emphasized with the potential or aim to restore the lubrication of cartilage to treat OA. However, there is no direct evidence that restoration of cartilage lubrication can reverse the pathologic progression of OA at its early stage (though it is most expected), so drug intervention should be taken into consideration especially at the middle or late stage of OA. Particularly, it should be noted that the animal models used in these studies are rats, the anatomic structure of the stifle joint of rats (the size of joint, the thickness of articular cartilage and the mechanical loading) are significantly different from humans, more clinically relevant data are required from the large animal models of OA (including dog, sheep/goat, and horse) to further evaluate the validities and potential therapeutics of such biomimetic lubricants.

## 4. Conclusions and Future Perspectives

The synergy between the phosphocholine lipids, HA and lubricin to form a highly hydrated boundary lubrication layer on the outer surface of cartilage is now generally accepted to explain the remarkable lubrication performance of articular cartilage, the integrity of this lubrication layer is vitally critical to maintaining the homeostasis of articular cartilage which is determined by the only embedded cells within it, chondrocytes. Otherwise, the initiation of OA is induced because of the increased friction of cartilage. Inspired by the molecular understanding of cartilage lubrication, the mechanosensitive nature of chondrocytes may give us the confidence to restore the impaired lubrication of articular cartilage at the early stage of OA to prevent the degradation of cartilage and promote the accumulation of the main components of ECM (type II collagen and aggrecan) to regenerate cartilage by regulating the metabolism of chondrocytes. It is, therefore, dual or multi-functional biomimetic lubricants (combined with drugs or inhibitors or gene regulators or autophagy) that may continuously attract attention in the future. 

## Figures and Tables

**Table 1:** The main biomechanical properties of the superficial zone, middle zone, and deep zone of articular cartilage.

Zones	Extracellular Molecules	Biomechanical Properties	References
Superficial zone (10 to 20%)	Outer of Surface Lubricin, HA, Phospholipids, COMP	Boundary lubrication, chondroprotection	[42,43,44]
	Below outer of Surface Type II collagen aggrecans, HA	Resist shear stress, bear ~20% load; Maintain tensile strength; As a barrier to fluid flow during loading; Subject to maximum strain; Contribute to elasticity and resiliency via interacting with collagen	[45,46,47]
Middle zone (40 to 60%)	Upper ~1/3rd Collagen type II, other collagens, aggrecans, HA	Transit shear and compression stresses; Exhibit high deformation during loading; Resist compression; Contribute to elasticity and resiliency to compression via interacting with collagen	[45,46,47,48]
	Lower ~2/3rd Thick collagen type II, other collagens, aggrecan, HA, GAGs	Compared with upper 1/3rd of the middle zone: Decrease tensile strength; Provide higher resistance to compression during loading	[49,50,51]
Deep zone (30 to 40%)	Thickest collagen type II, other collagens, aggrecans, HA, high GAGs	Relative to the middle zone: Further decreased tensile strength; Provide highest resistance to compression during loading	[49,50,51]

**Table 2:** Summary of typical biolubricants inspired by cartilage lubrication with the aim to treat OA.

System	Substrate or Tribopairs	Features	Comments	References
Natural lubricants and their derivatives				
HABpep	Human cartilage	Recruit free HA to resurface OA cartilage to decrease friction coefficients	Without further in vivo evaluation	[96]
HABP2-8-arm PEG-COLBP	C57/BL6 mice cartilage	Localize HA to cartilage surface	Reducing OA progression in young and aged mice	[97]
Peptide-modified chondroitin sulfate	Bovine cartilage	Resurface and reduce the friction coefficients without addition of HA	Without further in vivo evaluation	[98]
MPC grafted HA	Polystyrene microsphere versus silicon wafer	Upregulate anabolism and downregulate catabolism of chondrocytes in vitro	Lubrication was not estimated by cartilage; Without further in vivo evaluation	[99]
Catechol modified chitosan	Bovine cartilage	Recruit lubricin to reduce friction coefficients	Without further in vivo evaluation	[100]
Fully synthetic polymer lubricants				
PGA-LPMOXA-HBA	Bovine cartilage	Resurface the degraded cartilage via Schiff bases	Biocompatibility: Coated scaffold was implanted subcutaneously to check stability of formed film	[101]
PGA-CPMOXA-HBA	Bovine cartilage	Resurface the degraded cartilage via Schiff bases; Denser, more lubricious and biopassive films than PGA-LPMOXA-HBA	Anti-enzyme degradation; Without further in vivo evaluation	[102]
PAA-g-PEG	Bovine cartilage and rat cartilage	Anchor to cartilage surface by the ended thiol groups	Biocompatibility: No direct relation between lubrication and therapy was built in vivo by OA model of rats	[103,104,105]
Nanospheres or nanoparticles and nano (micro) gels				
PS-*g*-PSPMA nanospheres	Still ball versus Ti6Al4V alloy disk	Subsurface-initiated grafting polymer brush; Thick hydration layer	Low cytotoxicity; Without further in vivo evaluation	[106]
CS-PS nanoparticles	Polydimethylsiloxane ball versus Ti6Al4V	Couple hydration and rolling effect; Load anti-inflammation drug	Biocompatibility; Without further in vivo evaluation	[107]
MSNs-NH2@PMPC nanospheres	PE sphere pin versus Ti6Al4V disk	Enhance lubrication by hydration and sustained drug delivery	Biocompatibility; Evaluated by the rats OA model	[108]
MSNs@PSBMA nanoparticles	Polytetrafluoroethylene sphere versus Ti6Al4V sheet	Enhance lubrication by hydration and sustained drug delivery	Without further in vitro and in vivo evaluation	[109]
GelMA@DMA-MPC microgel	Polytetrafluoroethylene sphere versus silicon wafer	Enhance lubrication by hydration and sustained drug delivery	Biocompatibility; Evaluated by the rats OA model	[110]

## Data Availability

Not applicable.

## References

[1] Sophia Fox A.J., Bedi A., Rodeo S.A. (2009). The basic science of articular cartilage: Structure, composition, and function. Sports Health.

[2] Klein J. (2006). Molecular mechanisms of synovial joint lubrication. Proc. Inst. Mech. Eng. Part. J: J. Eng. Tribol..

[3] Kjirste C., Morrell W.A.H., David E., Krebs R.W. (2005). Mann Corrboration of in vivo cartilage pressures with implications for synovial joint tribology and osteoarthritis causation. Proc. Natl. Acad. Sci. USA.

[4] Marzo J.M., Gurske-DePerio J. (2009). Effects of medial meniscus posterior horn avulsion and repair on tibiofemoral contact area and peak contact pressure with clinical implications. Am. J. Sports Med..

[5] DeFrate L.E., Kim-Wang S.Y., Englander Z.A., McNulty A.L. (2019). Osteoarthritis year in review 2018: Mechanics. Osteoarthr. Cartil..

[6] Saberi Hosnijeh F., Bierma-Zeinstra S.M., Bay-Jensen A.C. (2019). Osteoarthritis year in review 2018: Biomarkers (biochemical markers). Osteoarthr. Cartil..

[7] Martinez-Moreno D., Jimenez G., Galvez-Martin P., Rus G., Marchal J.A. (2019). Cartilage biomechanics: A key factor for osteoarthritis regenerative medicine. Biochim. Biophys. Acta. Mol. Basis Dis..

[8] Morgese G., Benetti E.M., Zenobi-Wong M. (2018). Molecularly Engineered Biolubricants for Articular Cartilage. Adv. Healthc. Mater..

[9] Desrochers J., Amrein M.W., Matyas J.R. (2013). Microscale surface friction of articular cartilage in early osteoarthritis. J. Mech. Behav. Biomed. Mater..

[10] Lotz M.K., Carames B. (2011). Autophagy and cartilage homeostasis mechanisms in joint health, aging and OA. Nat. Rev. Rheumatol..

[11] Martel-Pelletier J., Barr A.J., Cicuttini F.M., Conaghan P.G., Cooper C., Goldring M.B., Goldring S.R., Jones G., Teichtahl A.J., Pelletier J.P. (2016). Osteoarthritis. Nat. Rev. Dis. Primers.

[12] Peter J Roughley J.S.M. (2014). The role of aggrecan in normal and osteoarthritic cartilage. J. Exp. Orthop..

[13] Linda Troeberg H.N. (2012). Proteases involved in cartilage matrix degradation in osteoarthritis. Biochim. Biophys. Acta.

[14] Liu-Bryan R., Terkeltaub R. (2015). Emerging regulators of the inflammatory process in osteoarthritis. Nat. Rev. Rheumatol..

[15] Sellam J., Berenbaum F. (2010). The role of synovitis in pathophysiology and clinical symptoms of osteoarthritis. Nat. Rev. Rheumatol..

[16] Mort J.S., Billington C.J. (2001). Articular cartilage and changes in arthritis matrix degradation. Arthritis Res. Ther..

[17] Bondeson J., Wainwright S., Hughes C., Caterson B. (2008). The regulation of the ADAMTS4 and ADAMTS5 aggrecanases in osteoarthritis: A review. Clin. Exp. Rheumatol..

[18] Brown S., Kumar S., Sharma B. (2019). Intra-articular targeting of nanomaterials for the treatment of osteoarthritis. Acta Biomater..

[19] Kloppenburg M., Berenbaum F. (2020). Osteoarthritis year in review 2019: Epidemiology and therapy. Osteoarthr. Cartil..

[20] Kwon H., Brown W.E., Lee C.A., Wang D., Paschos N., Hu J.C., Athanasiou K.A. (2019). Surgical and tissue engineering strategies for articular cartilage and meniscus repair. Nat. Rev. Rheumatol..

[21] Hulme C.H., Perry J., McCarthy H.S., Wright K.T., Snow M., Mennan C., Roberts S. (2021). Cell therapy for cartilage repair. Emerg. Top. Life Sci..

[22] Zamuner A., Cavo M., Scaglione S., Messina G.M.L., Russo T., Gloria A., Marletta G., Dettin M. (2016). Design of Decorated Self-Assembling Peptide Hydrogels as Architecture for Mesenchymal Stem Cells. Materials.

[23] Wieland H.A., Michaelis M., Kirschbaum B.J., Rudolphi K.A. (2005). Osteoarthritis—An untreatable disease?. Nat. Rev. Drug Discov..

[24] Arden N.K., Perry T.A., Bannuru R.R., Bruyere O., Cooper C., Haugen I.K., Hochberg M.C., McAlindon T.E., Mobasheri A., Reginster J.Y. (2021). Non-surgical management of knee osteoarthritis: Comparison of ESCEO and OARSI 2019 guidelines. Nat. Rev. Rheumatol..

[25] Goldberg V.M., Coutts R.D. (2004). Pseudoseptic reactions to hylan viscosupplementation: Diagnosis and treatment. Clin. Orthop. Relat. Res..

[26] Van der Weegen W., Wullems J.A., Bos E., Noten H., van Drumpt R.A. (2015). No difference between intra-articular injection of hyaluronic acid and placebo for mild to moderate knee osteoarthritis: A randomized, controlled, double-blind trial. J. Arthroplast..

[27] Jones E.S. (1936). Joint lubrication. Lancet.

[28] Bonnevie E., Bonassar L.J. (2020). A Century of Cartilage Tribology Research is Informing Lubrication Therapies. J. Biomech. Eng..

[29] He Z., Wang B., Hu C., Zhao J. (2017). An overview of hydrogel-based intra-articular drug delivery for the treatment of osteoarthritis. Colloids Surf. B Biointerfaces.

[30] Kuettner K., Aydelotte M., Thonar E. (1991). Articular cartilage matrix and structure: A minireview. J. Rheumatol. Suppl..

[31] Van der Kraan P., Buma P., Van Kuppevelt T., Van Den Berg W. (2002). Interaction of chondrocytes, extracellular matrix and growth factors: Relevance for articular cartilage tissue engineering. Osteoarthr. Cartil..

[32] Eyre D., Wu J., Woods P. (1991). The cartilage collagens: Structural and metabolic studies. J. Rheumatol..

[33] Eyre D. (1991). The collagens of articular cartilage. Semin. Arthritis Rheu..

[34] Eyre D.R., Wu J., Apone S. (1987). A growing family of collagens in articular cartilage: Identification of 5 genetically distinct types. J. Rheumatol..

[35] Bajpayee A.G., Grodzinsky A.J. (2017). Cartilage-targeting drug delivery: Can electrostatic interactions help?. Nat. Rev. Rheumatol..

[36] Kiani C., Chen L., Wu Y.J., Yee A.J., Yang B.B. (2002). Structure and function of aggrecan. Cell Res..

[37] Knudson W., Ishizuka S., Terabe K., Askew E.B., Knudson C.B. (2019). The pericellular hyaluronan of articular chondrocytes. Matrix Biol..

[38] Roughley P.J. (2006). The structure and function of cartilage proteoglycans. Eur. Cell Mater..

[39] Knudson C.B., Knudson W. (2001). Cartilage proteoglycans. Semin. Cell Dev. Biol..

[40] Melrose J., Smith S., Cake M., Read R., Whitelock J. (2005). Perlecan displays variable spatial and temporal immunolocalisation patterns in the articular and growth plate cartilages of the ovine stifle joint. Histochem. Cell Biol..

[41] Mow V.C., Ratcliffe A., Poole A.R. (1992). Cartilage and diarthrodial joints as paradigms for hierarchical materials and structures. Biomaterials.

[42] Raj A., Wang M., Liu C., Ali L., Karlsson N.G., Claesson P.M., Dėdinaitė A. (2017). Molecular synergy in biolubrication: The role of cartilage oligomeric matrix protein (COMP) in surface-structuring of lubricin. J. Colloid Interface Sci..

[43] Chang D.P., Guilak F., Jay G.D., Zauscher S. (2014). Interaction of lubricin with type II collagen surfaces: Adsorption, friction, and normal forces. J. Biomech..

[44] Kienle S., Boettcher K., Wiegleb L., Urban J., Burgkart R., Lieleg O., Hugel T. (2015). Comparison of friction and wear of articular cartilage on different length scales. J. Biomech..

[45] Quiroga J.P., Wilson W., Ito K., Van Donkelaar C. (2017). Relative contribution of articular cartilage’s constitutive components to load support depending on strain rate. Biomech. Modeling Mechanobiol..

[46] Henak C.R., Ross K.A., Bonnevie E.D., Fortier L.A., Cohen I., Kennedy J.G., Bonassar L. (2016). Human talar and femoral cartilage have distinct mechanical properties near the articular surface. J. Biomech..

[47] Kääb M.J., Ito K., Rahn B., Clark J.M., Nötzli H.P. (2000). Effect of mechanical load on articular cartilage collagen structure: A scanning electron-microscopic study. Cells Tissues Organs.

[48] Pierce D.M., Ricken T., Holzapfel G.A. (2013). A hyperelastic biphasic fibre-reinforced model of articular cartilage considering distributed collagen fibre orientations: Continuum basis, computational aspects and applications. Comput. Methods Biomech. Biomed. Eng..

[49] Halonen K., Mononen M., Jurvelin J., Töyräs J., Korhonen R. (2013). Importance of depth-wise distribution of collagen and proteoglycans in articular cartilage—A 3D finite element study of stresses and strains in human knee joint. J. Biomech..

[50] Venn M., Maroudas A. (1977). Chemical composition and swelling of normal and osteoarthrotic femoral head cartilage. I. Chemical composition. Ann. Rheum. Dis..

[51] Maroudas A., Venn M. (1977). Chemical composition and swelling of normal and osteoarthrotic femoral head cartilage. II. Swelling. Ann. Rheum. Dis..

[52] Romani P., Valcarcel-Jimenez L., Frezza C., Dupont S. (2020). Crosstalk between mechanotransduction and metabolism. Nat. Rev. Mol. Cell Biol..

[53] Li Y., Frank E.H., Wang Y., Chubinskaya S., Huang H.H., Grodzinsky A.J. (2013). Moderate dynamic compression inhibits pro-catabolic response of cartilage to mechanical injury, tumor necrosis factor-alpha and interleukin-6, but accentuates degradation above a strain threshold. Osteoarthr. Cartil..

[54] Fitzgerald J.B., Jin M., Grodzinsky A.J. (2006). Shear and compression differentially regulate clusters of functionally related temporal transcription patterns in cartilage tissue. J. Biol. Chem..

[55] Grodzinsky A.J., Levenston M.E., Jin M., Frank E.H. (2000). Cartilage tissue remodeling in reponse to mechanical forces. Annu. Rev. Biomed. Eng..

[56] Buschmann M.D., Gluzband Y.A., Grodzinsky A.J., Hunziker E.B. (1995). Mechanical compression modulates matrix biosynthesis in chondrocyte/agarose culture. J. Cell Sci..

[57] Yokota H., Goldring M.B., Sun H.B. (2003). CITED2-mediated regulation of MMP-1 and MMP-13 in human chondrocytes under flow shear. J. Biol. Chem..

[58] Mohtai M., Gupta M.K., Donlon B., Ellison B., Cooke J., Gibbons G., Schurman D.J., Smith R.L. (1996). Expression of Interleukin-6 in Osteosrthritic Chondrocytes and the Effects of Fluid-Induced Shear on This Expression in Normal Human Chondrocytes in Vitro. J. Orthop. Res..

[59] Abulencia J.P., Gaspard R., Healy Z.R., Gaarde W.A., Quackenbush J., Konstantopoulos K. (2003). Shear-induced cyclooxygenase-2 via a JNK2/c-Jun-dependent pathway regulates prostaglandin receptor expression in chondrocytic cells. J. Biol. Chem..

[60] Healy Z.R., Lee N.H., Gao X., Goldring M.B., Talalay P., Kensler T.W., Konstantopoulos K. (2005). Divergent responses of chondrocytes and endothelial cells to shear stress: Cross-talk among COX-2, the phase 2 response, and apoptosis. Proc. Natl. Acad. Sci. USA.

[61] Healy Z.R., Zhu F., Stull J.D., Konstantopoulos K. (2008). Elucidation of the signaling network of COX-2 induction in sheared chondrocytes: COX-2 is induced via a Rac/MEKK1/MKK7/JNK2/c-Jun-C/EBPbeta-dependent pathway. Am. J. Physiol. Cell Physiol..

[62] Wang P., Zhu F., Tong Z., Konstantopoulos K. (2011). Response of chondrocytes to shear stress: Antagonistic effects of the binding partners Toll-like receptor 4 and caveolin-1. FASEB J..

[63] Wang P., Guan P.P., Guo C., Zhu F., Konstantopoulos K., Wang Z.Y. (2013). Fluid shear stress-induced osteoarthritis: Roles of cyclooxygenase-2 and its metabolic products in inducing the expression of proinflammatory cytokines and matrix metalloproteinases. FASEB J..

[64] Lin W., Klein J. (2021). Recent Progress in Cartilage Lubrication. Adv. Mater..

[65] Waller K.A., Zhang L.X., Elsaid K.A., Fleming B.C., Warman M.L., Jay G.D. (2013). Role of lubricin and boundary lubrication in the prevention of chondrocyte apoptosis. Proc. Natl. Acad. Sci. USA.

[66] Bonnevie E.D., Delco M.L., Bartell L.R., Jasty N., Cohen I., Fortier L.A., Bonassar L.J. (2018). Microscale frictional strains determine chondrocyte fate in loaded cartilage. J. Biomech..

[67] McCutchen C.W. (1962). The frictional properties of animal joints. Wear.

[68] Lewis P., McCutchen C. (1960). Lubrication of mammalian joints. Nature.

[69] McCutchen C. (1959). Mechanism of animal joints: Sponge-hydrostatic and weeping bearings. Nature.

[70] Ateshian G.A. (2009). The role of interstitial fluid pressurization in articular cartilage lubrication. J. Biomech..

[71] Ateshian G., Wang H., Lai W. (1998). The role of interstitial fluid pressurization and surface porosities on the boundary friction of articular cartilage. J. Tribol..

[72] Schmidt T.A., Gastelum N.S., Nguyen Q.T., Schumacher B.L., Sah R.L. (2007). Boundary lubrication of articular cartilage—Role of synovial fluid constituents. Arthritis Rheum..

[73] Schmidt T.A., Sah R.L. (2007). Effect of synovial fluid on boundary lubrication of articular cartilage. Osteoarthr. Cartil..

[74] Banquy X., Lee D.W., Das S., Hogan J., Israelachvili J.N. (2014). Shear-Induced Aggregation of Mammalian Synovial Fluid Components under Boundary Lubrication Conditions. Adv. Funct. Mater..

[75] Lee D.W., Banquy X., Israelachvili J.N. (2013). Stick-slip friction and wear of articular joints. Proc. Natl. Acad. Sci. USA.

[76] Lin W., Liu Z., Kampf N., Klein J. (2020). The Role of Hyaluronic Acid in Cartilage Boundary Lubrication. Cells.

[77] Seror J., Merkher Y., Kampf N., Collinson L., Day A.J., Maroudas A., Klein J. (2011). Articular Cartilage Proteoglycans As Boundary Lubricants: Structure and Frictional Interaction of Surface-Attached Hyaluronan and Hyaluronan-Aggrecan Complexes. Biomacromolecules.

[78] Gaisinskaya-Kipnis A., Klein J. (2016). Normal and Frictional Interactions between Liposome-Bearing Biomacromolecular Bilayers. Biomacromolecules.

[79] Radin E.L., Swann D.A., Weisser P.A. (1970). Separation of a hyaluronate-free lubricating fraction from synovial fluid. Nature.

[80] Das S., Banquy X., Zappone B., Greene G.W., Jay G.D., Israelachvili J.N. (2013). Synergistic interactions between grafted hyaluronic acid and lubricin provide enhanced wear protection and lubrication. Biomacromolecules.

[81] Zappone B., Ruths M., Greene G.W., Jay G.D., Israelachvili J.N. (2007). Adsorption, lubrication, and wear of lubricin on model surfaces: Polymer brush-like behavior of a glycoprotein. Biophys. J..

[82] Hills B.A., Butler B.D. (1984). Surfactants indentified synovial fluid and their ability to act as boudary lubricants. Ann. Rheum. Dis..

[83] Sorkin R., Kampf N., Dror Y., Shimoni E., Klein J. (2013). Origins of extreme boundary lubrication by phosphatidylcholine liposomes. Biomaterials.

[84] Sorkin R., Kampf N., Zhu L., Klein J. (2016). Hydration lubrication and shear-induced self-healing of lipid bilayer boundary lubricants in phosphatidylcholine dispersions. Soft Matter.

[85] Jay G., Cha C. (1999). The effect of phospholipase digestion upon the boundary lubricating ability of synovial fluid. J. Rheumatol..

[86] Seror J., Zhu L., Goldberg R., Day A.J., Klein J. (2015). Supramolecular synergy in the boundary lubrication of synovial joints. Nat. Commun..

[87] Zhu L., Seror J., Day A.J., Kampf N., Klein J. (2017). Ultra-low friction between boundary layers of hyaluronan-phosphatidylcholine complexes. Acta Biomater..

[88] Pasquali-Ronchetti I., Quaglino D., Mori G., Bacchelli B., Ghosh P. (1997). Hyaluronan-Phospholipids interactions. J. Struct. Biol..

[89] Jahn S., Klein J. (2015). Hydration Lubrication: The Macromolecular Domain. Macromolecules.

[90] Klein J. (2013). Hydration lubrication. Friction.

[91] Jahn S., Seror J., Klein J. (2016). Lubrication of Articular Cartilage. Annu. Rev. Biomed. Eng..

[92] Kosinska M.K., Ludwig T.E., Liebisch G., Zhang R., Siebert H.-C., Wilhelm J., Kaesser U., Dettmeyer R.B., Klein H., Ishaque B. (2015). Articular Joint Lubricants during Osteoarthritis and Rheumatoid Arthritis Display Altered Levels and Molecular Species. PLoS ONE.

[93] Neu C.P., Reddi A.H., Komvopoulos K., Schmid T.M., Di Cesare P.E. (2010). Increased Friction Coefficient and Superficial zone Protein Expression in Patients With Advanced Osteoarthritis. Arthritis Rheum..

[94] Elsaid K.A., Jay G.D., Chichester C.O. (2007). Reduced expression and proteolytic susceptibility of lubricin/superficial zone protein may explain early elevation in the coefficient of friction in the joints of rats with antigen-induced arthritis. Arthritis Rheum..

[95] Antonacci J.M., Schmidt T.A., Serventi L.A., Cai M.Z., Shu Y.L., Schumacher B.L., McIlwraith C.W., Sah R.L. (2012). Effects of equine joint injury on boundary lubrication of articular cartilage by synovial fluid: Role of hyaluronan. Arthritis Rheum..

[96] Singh A., Corvelli M., Unterman S.A., Wepasnick K.A., McDonnell P., Elisseeff J.H. (2014). Enhanced lubrication on tissue and biomaterial surfaces through peptide-mediated binding of hyaluronic acid. Nat. Mater..

[97] Faust H.J., Sommerfeld S.D., Rathod S., Rittenbach A., Ray Banerjee S., Tsui B.M.W., Pomper M., Amzel M.L., Singh A., Elisseeff J.H. (2018). A hyaluronic acid binding peptide-polymer system for treating osteoarthritis. Biomaterials.

[98] Twitchell C., Walimbe T., Liu J.C., Panitch A. (2020). Peptide-Modified Chondroitin Sulfate Reduces Coefficient of Friction at Articular Cartilage Surface. Curr. Res. Biotechnol..

[99] Zheng Y., Yang J., Liang J., Xu X., Cui W., Deng L., Zhang H. (2019). Bioinspired Hyaluronic Acid/Phosphorylcholine Polymer with Enhanced Lubrication and Anti-Inflammation. Biomacromolecules.

[100] Wan H., Ren K., Kaper H.J., Sharma P.K. (2020). A bioinspired mucoadhesive restores lubrication of degraded cartilage through reestablishment of lamina splendens. Colloids Surf. B Biointerfaces.

[101] Morgese G., Cavalli E., Muller M., Zenobi-Wong M., Benetti E.M. (2017). Nanoassemblies of Tissue-Reactive, Polyoxazoline Graft-Copolymers Restore the Lubrication Properties of Degraded Cartilage. ACS Nano.

[102] Morgese G., Cavalli E., Rosenboom J.G., Zenobi-Wong M., Benetti E.M. (2018). Cyclic Polymer Grafts That Lubricate and Protect Damaged Cartilage. Angew Chem. Int. Ed..

[103] Samaroo K.J., Tan M., Andresen Eguiluz R.C., Gourdon D., Putnam D., Bonassar L.J. (2017). Tunable Lubricin-mimetics for Boundary Lubrication of Cartilage. Biotribology.

[104] Nemirov D., Nakagawa Y., Sun Z., Lebaschi A., Wada S., Carballo C., Deng X.H., Putnam D., Bonassar L.J., Rodeo S.A. (2020). Effect of Lubricin Mimetics on the Inhibition of Osteoarthritis in a Rat Anterior Cruciate Ligament Transection Model. Am. J. Sports Med..

[105] Samaroo K.J., Tan M., Putnam D., Bonassar L.J. (2017). Binding and lubrication of biomimetic boundary lubricants on articular cartilage. J. Orthop. Res..

[106] Feng H., Ma Z., Zhang Y., Liu F., Ma S., Zhang R., Cai M., Yu B., Zhou F. (2020). Polystyrene Nanospheres Modified with a Hydrophilic Polymer Brush through Subsurface-Initiated Atom Transfer Radical Polymerization as Biolubricating Additive. Macromol. Mater. Eng..

[107] Yang L., Zhao X., Zhang J., Ma S., Jiang L., Wei Q., Cai M., Zhou F. (2021). Synthesis of charged chitosan nanoparticles as functional biolubricant. J. Colloid Interface Sci. B Biointerfaces.

[108] Chen H., Sun T., Yan Y., Ji X., Sun Y., Zhao X., Qi J., Cui W., Deng L., Zhang H. (2020). Cartilage matrix-inspired biomimetic superlubricated nanospheres for treatment of osteoarthritis. Biomaterials.

[109] Wan L., Tan X., Sun T., Sun Y., Luo J., Zhang H. (2020). Lubrication and drug release behaviors of mesoporous silica nanoparticles grafted with sulfobetaine-based zwitterionic polymer. Mater. Sci. Eng. C.

[110] Han Y., Yang J., Zhao W., Wang H., Sun Y., Chen Y., Luo J., Deng L., Xu X., Cui W. (2021). Biomimetic injectable hydrogel microspheres with enhanced lubrication and controllable drug release for the treatment of osteoarthritis. Bioact. Mater..

[111] Rydell N.W., Butler J., Balazs E.A. (1970). Hyaluronic acid in synovial fluid. VI. Effect of intra-articular injection of hyaluronic acid on the clinical symptoms of arthritis in track horses. Acta Vet. Scand..

[112] Peyron J.G., Balazs E.A. (1974). Preliminary clinical assessment of Na-hyaluronate injection into human arthritic joints. Pathol. Biol..

[113] Chen M., Briscoe W.H., Armes S.P., Klein J. (2009). Lubrication at physiological pressures by polyzwitterionic brushes. Science.

[114] Wan H., Zhao X., Lin C., Kaper H.J., Sharma P.K. (2020). Nanostructured Coating for Biomaterial Lubrication through Biomacromolecular Recruitment. ACS Appl. Mater. Interfaces.

[115] Banquy X., Burdynska J., Lee D.W., Matyjaszewski K., Israelachvili J. (2014). Bioinspired Bottle-Brush Polymer Exhibits Low Friction and Amontons-like Behavior. J. Am. Chem. Soc..

[116] Faivre J., Shrestha B.R., Xie G., Olszewski M., Adibnia V., Moldovan F., Montembault A., Sudre G., Delair T., David L. (2018). Intermolecular Interactions between Bottlebrush Polymers Boost the Protection of Surfaces against Frictional Wear. Chem. Mater..

[117] Sun Z., Feeney E., Guan Y., Cook S.G., Gourdon D., Bonassar L.J., Putnam D. (2019). Boundary mode lubrication of articular cartilage with a biomimetic diblock copolymer. Proc. Natl. Acad. Sci. USA.

[118] Liu G., Cai M., Wang X., Zhou F., Liu W. (2016). Magnetite-Loaded Thermosensitive Nanogels for Bioinspired Lubrication and Multimodal Friction Control. ACS Macro Lett..

[119] Liu G.Q., Liu Z.L., Li N., Wang X.L., Zhou F., Liu W.M. (2014). Hairy Polyelectrolyte Brushes-Grafted Thermosensitive Microgels as Artificial Synovial Fluid for Simultaneous Biomimetic Lubrication and Arthritis Treatment. Acs Appl. Mater. Interfaces.

[120] Li Z., Ma S., Zhang G., Wang D., Zhou F. (2018). Soft/Hard-Coupled Amphiphilic Polymer Nanospheres for Water Lubrication. ACS Appl Mater. Interfaces.

